# Catalytic and hydrodynamic properties of styrene monooxygenases from *Rhodococcus opacus* 1CP are modulated by cofactor binding

**DOI:** 10.1186/s13568-015-0112-9

**Published:** 2015-06-04

**Authors:** Anika Riedel, Thomas Heine, Adrie H Westphal, Catleen Conrad, Philipp Rathsack, Willem J H van Berkel, Dirk Tischler

**Affiliations:** Interdisciplinary Ecological Center, Freiberg Environmental Microbiology Group, TU Bergakademie Freiberg, Leipziger Str. 29, 09599 Freiberg, Germany; Laboratory of Biochemistry, Wageningen University, Dreijenlaan 3, 6700ET Wageningen, The Netherlands; Institute of Analytical Chemistry, TU Bergakademie Freiberg, Leipziger Str. 29, 09599 Freiberg, Germany

**Keywords:** Flavoprotein, Monooxygenase, Oligomerization, FAD binding, *Rhodococcus opacus* 1CP, Styrene epoxidation

## Abstract

Styrene monooxygenases (SMOs) are flavoenzymes catalyzing the epoxidation of styrene into styrene oxide. SMOs are composed of a monooxygenase (StyA) and a reductase (StyB). The latter delivers reduced FAD to StyA on the expense of NADH. We identified *Rhodococcus opacus* 1CP as the first microorganism to possess three different StyA isoforms occurring in two systems StyA1/StyA2B and StyA/StyB, respectively. The hydrodynamic properties of StyA isozymes were found to be modulated by the binding of the (reduced) FAD cofactor. StyA1 and SyA2B mainly occur as dimers in their active forms while StyA is a monomer. StyA1 showed the highest epoxidation activity and excellent enantioselectivity in aromatic sulfoxidation. The hydrodynamic and biocatalytic properties of SMOs from strain 1CP are of relevance for investigation of possible industrial applications.

**Electronic supplementary material:**

The online version of this article (doi:10.1186/s13568-015-0112-9) contains supplementary material, which is available to authorized users.

## Introduction

Styrene monooxygenases (SMO; EC 1.14.14.11) are two-component flavoenzymes composed of a monooxygenase (StyA) and a FAD reductase (StyB) (Hartmans et al. 1990; Huijbers et al. 2014; Montersino et al. 2011; van Berkel et al. 2006). StyB releases electrons from NADH to oxidized FAD which is then translocated in its reduced state to StyA. Incorporation of reduced FAD in StyA and subsequent reaction with molecular oxygen yields flavin hydroperoxide, which stimulates the binding of styrene and its subsequent epoxidation (Kantz and Gassner 2011).

SMOs have been extensively characterized with respect to the enantioselective conversion of styrene derivatives (Panke et al. 2000; Park et al. 2006a, b; Tischler et al. 2009). The first SMOs studied originated from bacterial isolates of various soil samples (Hartmans et al. 1990), followed by SMOs from *Pseudomonas fluorescens* ST (Beltrametti et al. 1997; Gennaro et al. 1999; Marconi et al. 1996), *Pseudomonas* spp. Y2 (Velasco et al. 1998) and VLB120 (Hollmann et al. 2003; Otto et al. 2004), *Pseudomonas putida* S12 (Kantz et al. 2005; Morrison et al. 2013; Ukaegbu et al. 2010), *Pseudomonas putida* CA-3 (Nikodinovic-Runic et al. 2013; O’Conner et al. 1995), metagenome screening (van Hellemond et al. 2007), and *Rhodococcus* spp. ST-5 and ST-10 (Toda et al. 2012; Toda and Itoh 2012).

**Figure 1:**
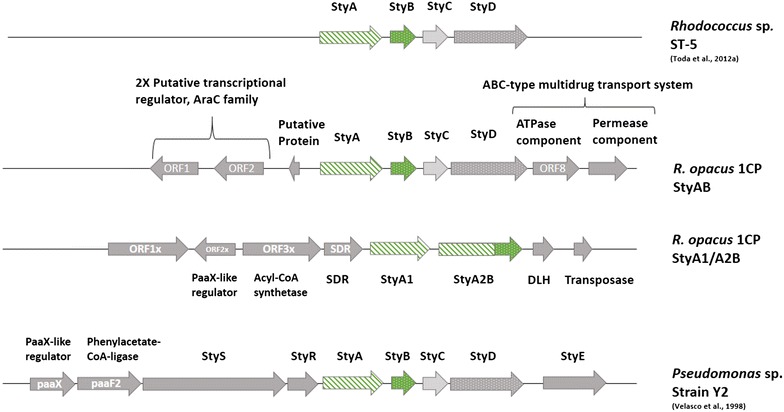
Structural organization of StyA/StyB- and StyA1/StyA2B-gene clusters from *Rhodococcus opacus* 1CP. The *styABCD* cluster of strain 1CP harboring a styrene monooxygenase (StyA) and the associated oxidoreductase (StyB), as well as the styrene oxide isomerase (StyC) and the phenylacetaldehyde dehydrogenase (StyD) (Oelschlägel et al. 2014). Upstream two putative transcriptional regulators (AraC family) are shown. *Rhodococcus* sp. ST-5 harbors a similar cluster (Toda and Itoh 2012). The StyA1/StyA2B system of strain 1CP containing the styrene monooxygenase StyA1 and the self-sufficient StyA2B where the StyA part is fused to the StyB subunit. ORF2x directly upstream from the genes responsible for conversion of styrene encodes a PaaX-like regulator. This regulator is responsible for activation of the styrene upper pathway metabolism product phenylacetic acid, which is then converted in their CoA-associated product in order to be available for the Krebs cycle. *SDR* short-chain dehydrogenase, *DLH* dienelactone hydrolase (Patrauchan et al. 2005; Tischler et al. 2012). *Pseudomonas* sp. Y2 exhibits a *styABCD* cluster and the PaaX-like regulator. In comparison to rhodococci *Pseudomonas* species feature two histidine sensor kinases StyS and StyR.

**Figure 2:**
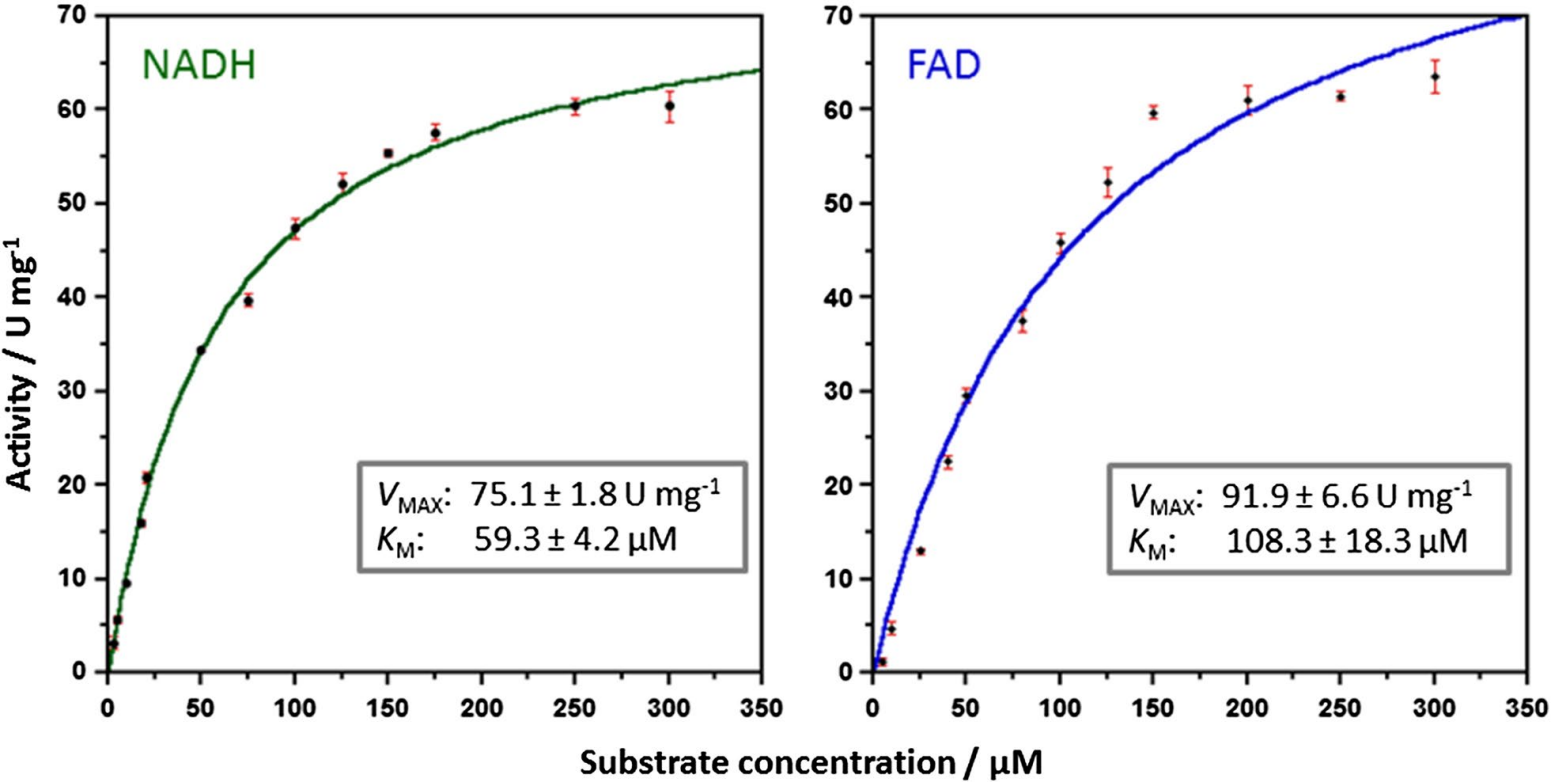
Michaelis–Menten kinetics of StyB from *Rhodococcus opacus* 1CP. The purified reductase was applied to enzyme assays as described in “Materials and methods” section. By varying the concentration of one of the substrates (NADH *left panel* or FAD *right panel*) apparent *V*_MAX_ and *K*_M_-values were obtained by fitting initial velocities to the Michaelis–Menten equation. Data points shown are mean values of at least triplicate measurements and standard errors for each data-set are shown as *red bars.*

The first one-component SMO (StyA2B) was discovered from *Rhodococcus opacus* 1CP representing a natural fusion between the oxygenase and reductase subunits (Tischler et al. 2009). The epoxidase activity of StyA2B appeared to be rather low. However, a second oxygenase (StyA1), present in the same gene cluster, boosted the epoxidase activity when StyA2B was used as partner reductase (Tischler et al. 2010). More recently, we found another SMO cluster in the *R. opacus* 1CP genome (KF540256) (Oelschlägel et al. 2014). This *styABCD* cluster is similar to those of pseudomonads and *Rhodococcus* sp. ST-5. Remarkably, this discovery makes *R. opacus* 1CP the first organism possessing both one- and two-component SMOs (Figures 1, 2) raising the question about the evolution of the two types of SMOs.

SMO oxygenase and reductase components usually occur as homodimers (Morrison et al. 2013; Otto et al. 2004; Tischler et al. 2010; Ukaegbu et al. 2010). StyAs from *Rhodococcus* sp. ST-5 and ST-10 were recently reported to be in monomer–dimer equilibrium (Toda et al. 2012). However, these proteins were studied in their apo-form and the effect of flavin binding was not considered. Because oligomerization might influence the catalytic efficiency of SMO systems (Otto et al. 2004), we here address the hydrodynamic properties of StyA1, StyA2B and StyA from *R. opacus* 1CP in the absence and presence of oxidized or reduced FAD. The results obtained are discussed in relation to the catalytic and structural features of SMOs.

## Materials and methods

### Chemicals

Styrene, styrene oxide, phenyl vinyl sulfide, phenyl vinyl sulfoxide, and cofactors were purchased from Sigma-Aldrich (Steinheim, Germany) and Carl Roth (Karlsruhe, Germany). Restriction enzymes were received from MBI Fermentas (St. Leon-Rot, Germany) and New England Biolabs GmbH (Frankfurt am Main, Germany). Oligonucleotides and synthetic genes were synthesized by Eurofins MWG Operon (Ebersberg, Germany).

### Bacterial strains, plasmids, and culture conditions

Bacterial strains and plasmids used in this study were treated according to Tischler et al. (2009). Other plasmids and primers are listed in Table 1. *Escherichia coli* BL21 strains were grown aerobically in LB-media (100 µg mL^−1^ ampicillin, and 50 µg mL^−1^ chloramphenicol) at 37°C while shaking constantly at 120 rpm in baffled flasks.

**Table 1:** Plasmids and primers used in this study

Plasmid or primer	Relevant characteristic(s)	Source
Plasmids		
pSRoA_P01	*styA* of *R. opacus* 1CP (1.281-kb *Nde*I/*Kpn*I fragment) cloned into pET16bP	This study
pSRoB_P01	*styB* of *R. opacus* 1CP (545-kb *Nde*I/*Not*I fragment) cloned into pET16bP	This study
pSPfB_P01	*styB* of *Pseudomonas fluorescens* ST (510-kb *Kpn*I fragment) cloned into pET16bp	This study
pJET1.2/blunt	Contains a gene coding for a lethal restriction enzyme; a gene disrupted by ligation of a DNA insert into the cloning site is possible; T7 promoter	Thermo Scientific
pET16bp	pET16b with additional multicloning site; allows expression of recombinant proteins with N-terminal His_10_-tag	U. Wehmeyer*
pEX_A_StyB	pEX vector with additional multiple cloning site; (Amp^r^)	Eurofins MWG Operon
Primers		
1CP_A_fw	5′-CATATGAGCAAGCGAATC-3′; includes *Nde*I site	This study
1CP_A_rev	5′-GGTACCTCATGGCTGTGC-3′; includes *Kpn*I site	This study
ST_B_fw	5′-GGTACCATATGACGTTAAAAAAAGATGTG-3′; includes *Kpn*I site	This study
ST_B_rev	5′-GGTACCTTAGTTCAGCGGCAACGGCTT G-3′; includes *Kpn*I site	This study

* Personal communication; Tischler et al. (2009).

The strains *Rhodococcus opacus* 1CP (DSMZ; DSM 46757, and VKM; Ac-2638) and *Pseudomonas fluorescens* ST (DSMZ; DSM 6290) are available from public culture collections.

### Construction of expression clones

The *styA* gene was amplified from genomic *R. opacus* 1CP DNA by PCR (annealing temperature ≙ 58.6°C) by applying the appropriate primers (Table 1). The purified products were cloned into pJET1.2/blunt cloning vector using the CloneJET PCR Cloning Kit (Thermo Scientific). To yield the expression construct pSRoA_P01, *styA* was double digested (*Nde*I/*Kpn*I) and ligated into similarly treated pET16bp.

The *styB* gene originating from strain 1CP was codon optimized (accession number: KP711388) and cloned in a pEX-A vector system flanked by the restriction sites *Nde*I and *Not*I. The GC content of *styB* was adapted to the codon usage of strain *Acinetobacter* sp. ADP1 allowing for higher gene expression levels (with *E*. *coli* BL21 or alternatively with *Acinetobacter* species as host) yielding soluble protein. The received vector was used in analogy to the *styA* cloning procedure to yield a *styB* construct of pET16bp designated as pSRoB_P01.

Another *styB* gene was amplified from *Pseudomonas fluorescens* ST (Beltrametti et al. 1997) by PCR (annealing temperature ≙ 54.8°C) by applying the appropriate primers (Table 1). Products obtained were cloned into pJET1.2/blunt cloning vector as described above. To yield the expression construct pSPfB_P01, *styB* was digested (*Kpn*I) and subsequently ligated into similarly treated pET16bp.

### Gene expression, protein purification and storage

Recombinant proteins were obtained as His_10_-tagged fusion proteins. The *styA*, *styA1* and *styA2B* expression took place in a 5-L biofermentor as described previously (Tischler et al. 2009, 2010), or in 2-L flasks, respectively. When cell density reached an OD_600_ of 0.5, induction was started by addition of 0.05 mM IPTG (isopropyl-β-d-thiogalactopyranoside) (120 rpm, 22 h, 20°C). Cells were harvested by centrifugation (5,000×*g*, 30 min, 4°C), resuspended in 10 mM Tris–HCl (pH 7.5), and stored at −80°C. The *styB* expression was performed under similar conditions as described for *styA’s*. Expression was highly improved when LB media contained 0.5 M sodium chloride, 2.5 mM betaine, and 0.2% glucose, yielding soluble StyB. For separation of soluble StyB from unsoluble matter, the suspension was centrifuged for 2 h.

Purification of StyA, StyA1, StyA2B and StyB was performed on 1-mL HisTrap FF columns (Tischler et al. 2009; Thiel et al. 2005), avoiding dithiothreitol (DTT) in all buffers. Purified StyA proteins were precipitated with ammonium sulfate (80% saturation) or concentrated via Amicon Ultra Centrifugal Filters (Regenerated Cellulose 10,000 MWCO, Millipore), and resuspended in 10 mM Tris–HCl (pH 7.5), respectively. Purified StyB was concentrated via Amicon Ultra Centrifugal Filters in the presence of 10 µM FAD at 4°C. In order to remove imidazole and excess salt, proteins were treated with a 10 mL gravity-flow gel filtration column (Econo Pac® 10DG Desalting Column, Bio-Rad) running in 20 mM Tris–HCl (pH 7.5). Proteins were stored at −20°C in 10 mM Tris–HCl (pH 7.5), containing 50% [v/v] glycerol.

Expression, purification, and refolding of StyB originating from *Pseudomonas fluorescens* ST was performed as described previously for StyB from *Pseudomonas* sp. VLB120 (Otto et al. 2004; Tischler et al. 2010) and thereof StyB was obtained as apoprotein.

Apo-forms of StyA, StyA1 and StyA2B were prepared on a HisTrap FF column at pH 7.5. Washing the protein bound to the column with buffer (10 mM Tris–HCl, 500 mM NaCl; pH 7.5) completely removed the weakly bound FAD.

### SMO activity and protein content determination

Reductase activity of recombinant StyA2B and StyB’s was quantified spectrophotometrically by determination of NADH consumption at 340 nm (ɛ = 6.22 mM^−1^ cm^−1^, Otto et al. 2004) (SpectraMax M2e, Molecular Devices). The standard assay was arranged in a 96-well plate (total volume of each well was 300 µL) containing 8 mM Tris–HCl (pH 7.5), 175 µM NADH, 60 µM FAD, and an appropriate amount of recombinant StyA2B or StyB, respectively. After 10 min of pre-incubation at 30°C, the reaction was started by adding NADH. Checkpoints were taken at 2 s interval. Initial reaction rates were measured using 2–300 µM FAD or NADH while keeping the corresponding co-substrate at a constant concentration in excess. In case of StyB from strain 1CP higher concentrations of substrates were necessary and therefore the NADH consumption was determined at 320 nm (ɛ = 4.65 mM^−1^ cm^−1^).

Monooxygenase activity of recombinant StyA, StyA1, and StyA2B with styrene or phenyl vinyl sulfide was determined via quantification of the products styrene oxide or phenyl vinyl sulfoxide, respectively. The enzymatic assay and standard HPLC analytic were performed as described previously (Tischler et al. 2009). The protocol was modified in order to determine the sulfide and sulfoxide via HPLC. Isocratic elution of respective compounds occurred with a 40% methanol/water eluent at a flow rate of 0.7 mL min^−1^. Products obtained were analyzed for enantiomers formed according to previously performed methods for epoxides (Tischler et al. 2009) or for sulfoxides (Anderson et al. 2002).

Protein content was determined using BCA Protein Assay Reagent (Thermo Scientific) or with the Bradford method using Protein Assay Reagent (Bio-Rad), respectively. Bovine serum albumin (Sigma) was used as a standard. Purity of protein batches was controlled by SDS-PAGE analysis (see Additional file 1). A purity of 95% or higher was found for all proteins even of various expression attempts of same protein. The purity of these batches was considered in calculating the activity values and for analytical gel filtration.

### Analytical gel filtration

The hydrodynamic properties of SMOs were analyzed on an Äkta_*explorer*_ FPLC system (Pharmacia Biotech) applying a Superdex 200 HR 10/30 column (bed volume V_t_ = 22.0 mL, GE Healthcare Life Sciences). The flow rate was 0.6 mL min^−1^ and the temperature was kept at 22°C. 100 µL of sample solution (~2 mg mL^−1^ protein of 95% or higher purity) was separated by applying a mobile phase containing 10 mM Tris–HCl (pH 7.2), and 500 mM sodium chloride. The elution behavior of oxidized holoenzymes was studied by adding 12.7 µM FAD to both buffer and sample solution. For studying the elution behavior of the reduced holoenzymes, 1 mM sodium dithionite was applied to all FAD-containing solutions. For the latter the FPLC system was extensively washed with anaerobic buffer containing 1 mM sodium dithionite. The identity of the eluted proteins was checked by analysis of collected fractions on SDS-PAGE.

The apparent molecular masses (*M*_r_) of SMOs were determined from running the following calibration proteins under similar conditions: myoglobin (17.8 kDa, 17.3 mL), chymotrypsin (25 kDa, 17.1 mL), ovalbumin (42.8 kDa, 15.3 mL), and bovine serum albumin (68 kDa, 14.3 mL and 136 kDa, 12.5 mL). Dextran blue (2,000 kDa) was used to determine the void volume (V_0_ = 7.9 mL). Apparent *M*_r_ values of SMOs were obtained from a graph where the partition coefficients (*K*_av_) of the standard proteins were plotted against log *M*_r_:

$$M_{r} = 10^{{\frac{{K_{av} - 1,1884}}{ - 0.4035}}} .$$

## Results

### Identification of a novel two-component styrene monooxygenase from *Rhodococcus opacus* 1CP

The discovery of the *styA2B* gene in a cluster with *styA1* (Tischler et al. 2009) prompted the search of other styrene catabolic genes in *R. opacus* 1CP. Interestingly, a third SMO gene *styA* was found within a *styABCD* cluster (accession number: KF540256) (Figure 1) of the recently completed genome sequence of *R. opacus* 1CP (Oelschlägel et al. 2014). Next to the *styA* gene, the *styABCD* cluster harbors genes for a flavin reductase (*styB*), as well as styrene oxide isomerase (*styC*) and phenylacetaldehyde dehydrogenase (*styD*). A highly related gene cluster is present in *Rhodococcus* sp. ST-5 (Figure 1) (Toda and Itoh 2012). Further, similar chromosomal regions have been described for *Pseudomonas fluorescens* ST (Beltrametti et al. 1997), *Pseudomonas putida* SN1 (Park et al. 2006a, b), *Pseudomonas* sp. Y2 (Velasco et al. 1998), as well as *Pseudomonas* sp. VLB120 (Panke et al. 1998).

The s*tyA* and *styB* genes from *R. opacus* 1CP encode for proteins with 427 and 178 amino acid residues with calculated molecular masses of 46,580 and 19,053 Da, respectively. The deduced amino acid sequence of StyA showed high identity to StyA from *Rhodococcus* sp. ST-5 (81%), and ST-10 (73%), as well as to StyA proteins from pseudomonads (~59%). However, the identity to StyA1 and the oxygenase part of StyA2B (aa 1–413) is only 28–29% in both cases.

The deduced amino acid sequence of flavin reductase StyB showed most identity with StyB from *Rhodococcus* sp. ST-5 (81%) and ST-10 (62%), and moderate identity with StyBs from pseudomonads (44–47%). The identity to the reductase part of StyA2B (aa 414–573; 160 aa) is rather low (25%).

In summary, a novel two-component styrene monooxygenase was identified in *R. opacus* 1CP that shares high sequence identity and gene cluster organization to the corresponding systems from *Rhodococcus* sp. ST-5 and to those of pseudomonads (Figure 1).

### Overexpression and purification of StyA and StyB

The *styA* and *styB* genes from *R. opacus* 1CP were successfully cloned into pET16bp and transformed into *E. coli* BL21. When expression was successful, a blue staining could be observed due to formation of indigo from indole originating from tryptophan. From *styA* expression, a cell dry weight of 1.2 g and 7.8 mg of soluble StyA protein per liter culture was obtained.

StyB was produced by expression of the BL21 cells in high salt LB-media (0.5 M NaCl) containing extra 2.5 mM betaine and 0.2% glucose. From *styB* expression, a cell dry weight of 1.4 g and 17.4 mg soluble StyB protein per liter culture was obtained.

Recombinant His_10_-tagged StyA and StyB proteins were purified via immobilized metal ion affinity chromatography (IMAC). Collected fractions were analyzed by SDS-PAGE showing that StyA as well as StyB are highly pure after the described procedure (see Additional file 1). Recombinant StyA possesses an apparent subunit molecular mass of 50 kDa including the His-tag while StyB exhibits an apparent subunit molecular mass of 21.5 kDa, in agreement with the deduced gene sequences.

StyA1 and StyA2B were obtained from expression attempts and protein purification as described earlier (Tischler et al. 2009, 2010). Purity was controlled by SDS-PAGE prior further analysis and application in biotransformation.

StyB from *R. opacus* 1CP was obtained in soluble form, which is rather unusual for StyB-homologs (Otto et al. 2004; Yeo et al. 2009). Maximum activities of 75.1 ± 1.8 and 91.9 ± 6.6 U mg^−1^, and *K*_M_ values of 59.3 ± 4.2 and 108.3 ± 18.3 µM were determined for the substrates NADH and FAD, respectively (Figure 2; Table 2).

**Table 2:** Kinetic parameters of Sty(A2)B reductases

Reductase-strain	Substrate changed*	Maximum activity (U mg^−1^)	*k*_cat_ (s^−1^)	*K*_M_ (µM)	Catalytic efficiency (s^−1^ µM^−1^)
StyB-1CP	NADH	75.1 ± 1.8	26.9 ± 0.6	59.3 ± 4.2	0.454
	FAD	91.9 ± 6.6	32.9 ± 2.3	108.3 ± 18.3	0.304
StyB-ST	NADH	28.8	8.8 ± 0.1	18.9 ± 1.0	0.466
	FAD	28.7	8.7 ± 0.3	2.6 ± 0.4	3.346
StyA2B-1CP**	NADH	3.7	3.9 ± 0.3	58 ± 9	0.068
	FAD	4.9	5.2 ± 0.2	26 ± 2	0.203

* One of the substrates was applied in excess and the concentration of the other one was varied.

** Data as previously reported (Tischler et al. 2010).

StyB from *R. opacus* 1CP was found to be unstable in time and not suitable for SMO epoxidation experiments. For that purpose (vide infra), we used StyB from *P. fluorescens* ST. StyB from strain ST was successfully refolded from inclusion bodies to give a highly pure and active FAD reductase (Table 2). StyB from strain ST showed maximum activities of 28.8 ± 0.4 and of 28.7 ± 1.0 U mg^−1^, and *K*_M_-values of 18.9 ± 1.0 and of 2.6 ± 0.4 µM for the substrates NADH and FAD, respectively. The catalytic efficiency of StyB from strain ST is comparable to that of StyB from strain 1CP, mainly because of a better affinity for both NADH and FAD (Table 2).

### StyA1 is the most active styrene monooxygenase from strain 1CP

Oxygenase activities of StyA1, StyA2B and StyA were determined by measuring the product of styrene oxidation (styrene oxide) via HPLC. StyB reductase, formate, formate dehydrogenase and catalase were added in order to produce reduced FAD, regenerate NADH, and remove hydrogen peroxide. All StyA enzymes were incubated with a threefold excess of StyB from *P. fluorescens* ST over StyAs.

From these experiments StyA1 showed a specific styrene epoxidation activity of 0.12 ± 0.02 U mg^−1^ while StyA and the fusion protein StyA2B revealed specific activities of 0.08 ± 0.01 and 0.037 ± 0.01 U mg^−1^, respectively. StyAs from *Rhodococcus* ST-5 (0.03 U mg^−1^) and ST-10 (0.026 U mg^−1^) were reported to possess lower activities (Toda et al. 2012). These findings state StyA1 and StyA from *R. opacus* 1CP as the most active SMOs from rhodococci so far.

**Figure 3:**
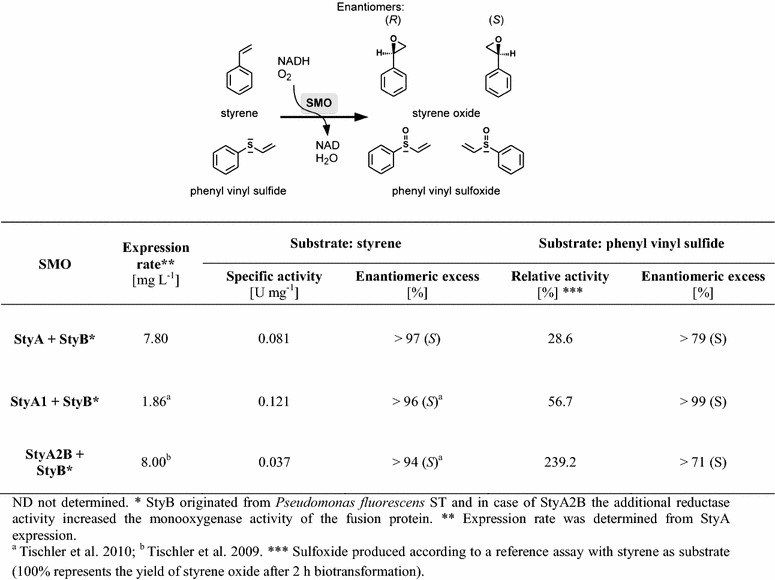
Specific activities and enantioselectivities of SMOs from *R. opacus* 1CP

Product analysis confirmed an earlier finding (Tischler et al. 2010) that StyA1 and StyA2B are able to epoxidize styrene to the (*S*)-enantiomer with an *ee* of 94%. The newly discovered StyA catalyzed the formation of this enantiomer with an *ee* exceeding 97% (Figure 3).

Besides epoxidation also the sulfoxidation was assayed. The substrate chosen was phenyl vinyl sulfide in order to provide a sulfoxidation as well as an epoxidation site (Figure 3). All three SMO isoforms were found to convert this substrate and only sulfoxidation was determined. The activities are comparable to styrene epoxidation. Notably, StyA2B showed a 2.4-times higher sulfoxidase as epoxidase activity. All three SMO isoforms were found to catalyze the sulfoxidation in an enantioselective manner. However, StyA1 stood out by producing more than 99% *S*-enantiomer of phenyl vinyl sulfoxide.

### Quaternary structure of SMO systems

**Figure 4:**
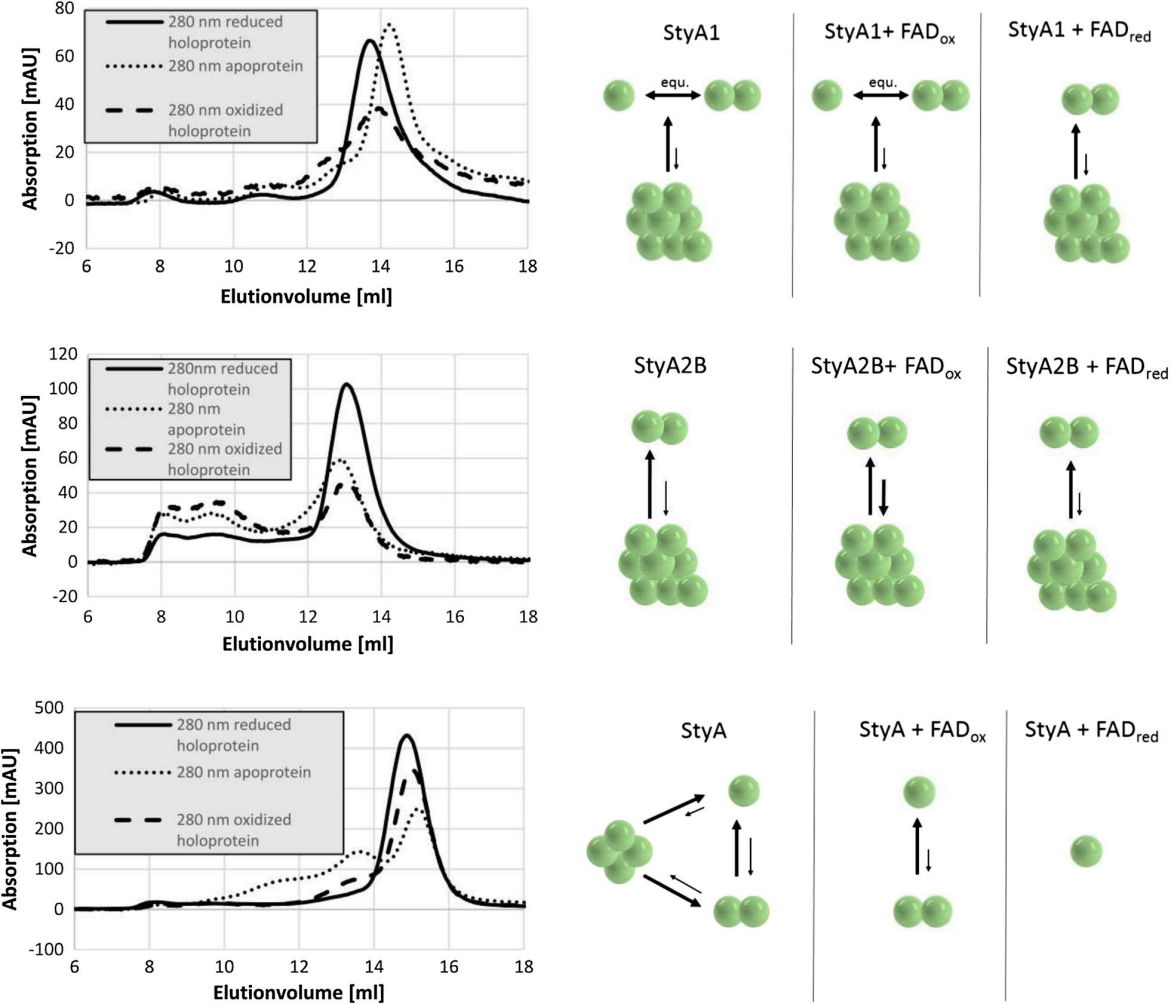
Size exclusion chromatography on Superdex-200 of apo-, holo- and reconstituted StyAs. *Left* Samples of 100 µL protein (1.95 mg mL^−1^ StyA1, 2.17 mg mL^−1^ StyA2B, 11.45 mg mL^−1^ StyA) were loaded onto the column. Elution was performed in 10 mM Tris–HCl (pH 7.2), 500 mM sodium chloride, pH 7.2 at 22°C. The flow rate was 0.6 mL min^−1^. Reconstituted enzymes were prepared by incubating both protein and buffer with 12.7 µM FAD, either in the absence or presence of 1 mM sodium dithionite. *Right* Distribution of the isoforms in the three conditions: apo, holo and reduced StyA. Intensity of *arrows* shows dominant direction of equilibrium (compare with Table 3).

Figure 4 shows the elution behavior of apo-, holo-, and reconstituted StyA1 on a Superdex200 gel filtration column. For the apoenzyme the main fraction eluted around 14.3 mL, while smaller fractions eluted around 13.0, 11.1 and 8.2 mL. This suggests that under the conditions applied, apo-StyA1 mainly occurs as a monomer, in equilibrium with dimers and higher-order quaternary forms. For holo-StyA1, the equilibrium shifted towards the dimeric form. When holo-StyA1 was fully reduced, the enzyme was almost exclusively present as a dimer (13.8 mL) and only a small fraction of oligomers remained.

StyA2B formed dimers as well as larger species (Figure 4). For both apo- and holo-StyA2B considerable amounts of higher-order quaternary forms were observed. Under reduced conditions, a clear shift into the direction of dimers (12.9 mL) occurred.

A strikingly different hydrodynamic behavior was observed for StyA (Figure 4). StyA mainly was found as a monomer (15.2 mL) under the conditions applied. In the apo-state approximately 10% tetramer (11.5 mL) and 30% dimer (13.7 mL) was found next to the monomer. When FAD was added, the amount of monomer increased to 80%, while the tetramer no longer existed. In the reduced form, holo-StyA was almost completely present as a monomer. The elution volume of the monomer decreased upon binding FAD_ox_ and FAD_red_ resulting in an apparent molecular mass shift of about 2–4 kDa. This shift arises probably due to the stronger binding of reduced FAD shifting the fast monomer–dimer equilibrium present in the apo form towards the pure monomer form resulting in a somewhat larger elution volume.

StyB from *Rhodococcus opacus* 1CP was found to occur as dimer in the presence of the cofactor FAD (not shown). Due to its low stability, no data were collected for the holoprotein under reduced conditions.

## Discussion

We found that the Gram-positive actinobacterium *Rhodococcus opacus* 1CP contains three different SMOs. Next to the previously reported *styA1* and *styA2B* genes (Tischler et al. 2009), the third SMO gene is located within a *styABCD* cluster, completely separate from *styA1* and *styA2B*. The *styABCD* cluster of *R. opacus* 1CP differs from styrene clusters of *Pseudomonas* species (Figure 1). The latter clusters exhibit a pathway-specific regulatory apparatus of two sensor kinases StyS and StyR as well as a transcriptional repressor PaaX (Panke et al. 1998; Velasco et al. 1998; Yeo et al. 2009). Similar sensor kinases are lacking in *R. opacus* 1CP as well as in *Rhodococcus* sp. ST-5 (Toda and Itoh 2012), but a PaaX-like regulator is present upstream from *styA1/styA2B* in strain 1CP (Tischler et al. 2009). Thus, in strain 1CP, both the *styA1/styA2B* gene cluster and the newly discovered *styABCD*-cluster might be regulated differently compared to that found for *Pseudomonas* species (Alonso et al. 2003; Oelschlägel et al. 2014). The presence of three StyA isoenzymes in a single strain is new and underlines the gene redundancy of rhodococci (Gröning et al. 2014a; Patrauchan et al. 2005). The different gene cluster organization as well as the respective regulatory machinery might indicate a convergent evolution (Tischler et al. 2012).

The newly discovered FAD reductase StyB from strain 1CP was found to be highly active with NADH as electron donor. However, in comparison to StyB-homologs (Otto et al. 2004; Tischler et al. 2009; Toda et al. 2012), the binding affinity towards NADH and FAD is rather low. StyB forms a homodimer, as reported for other StyB enzymes (Gröning et al. 2014b; Otto et al. 2004; Toda et al. 2012), but appeared to be rather unstable, limiting its further characterization.

StyA enzymes become active after receiving a reduced FAD molecule from their StyB partner. The tightly bound reduced flavin then reacts with molecular oxygen yielding a flavin hydroperoxide that acts as oxygen donor in the subsequent epoxidation reaction (Kantz and Gassner 2011). Here, by using StyB from *P. fluorescens* ST as partner reductase, we show that the oxygenation activity of StyA is in between that of StyA1 and StyA2B (Figure 3) and higher than that of StyA enzymes from other rhodococci. The StyA isoforms convert phenyl vinyl sulfide specifically into the corresponding sulfoxide with a similar activity as with styrene. Especially StyA1 shows excellent enantioselectivity in the sulfoxidation reaction by producing more than 99% of the *S*-enantiomer. With all three StyA isoforms no over-oxidation and no epoxidation of the adjacent vinyl chain was observed. This is important for biocatalytic applications and indicates that the vinyl moiety of phenyl vinyl sulfide is bound in the StyA active sites remote from the flavin hydroperoxide.

The successful combination of StyA enzymes from *R. opacus* 1CP with a foreign FAD reductase supports a diffusible transfer mechanism of reduced FAD (Hollmann et al. 2003; Kantz et al. 2005; Morrison et al. 2013). Here, we obtained evidence that such a mechanism can occur with both monomeric and dimeric StyA forms. After purification, the StyA isoforms occurred in their apo-form. Analytical gel filtration revealed that the hydrodynamic properties of the isoforms change in the presence of (reduced) FAD. StyA1 and StyA2B constitute dimers under reduced conditions while StyA is a monomer. This shows for the first time that StyA oxygenases can be active as monomers.

**Figure 5:**
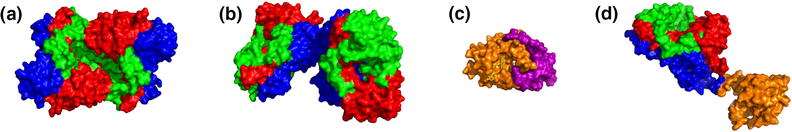
Schematic view on possible dimer structures of styrene monooxygenase components. StyA has the same fold as PHBH and the domains were accordingly *colored* (Montersino et al. 2011, 2013; Schreuder et al. 1988). FAD binding domain in *green*, substrate binding domain in *red*, and dimer interface in *blue*. The three-dimensional structure of StyA from *Pseudomonas* sp. S12 (PDB ID: 3IHM, Ukaegbu et al. 2010) is shown in **a**, where the active site clefts of monomers point to the center and no PHBH-like dimer was determined. A modelled StyA-dimer is presented in **b**. Here the dimer interfaces interact in a flavoprotein hydroxylase mode (Montersino et al. 2013; Schreuder et al. 1988) and a ‘back to back’ situation is shown in which active sites point into the medium. The experimentally solved three-dimensional StyB-structure (PDB ID: 4F07, Morrison et al. 2013) is shown as dimer in (**c)**. Together the structures of **a** and **c** were used to generate a model of StyA2B (**d**) in which two dimer interfaces occur (van den Heuvel et al. 2004). For clarity, only one StyA2B monomer is shown.

Our data clearly indicate that StyA1 is a dimer in its active form. This raises the question about how the dimer interface of this enzyme is formed. StyA1 might form a ‘back to back’ dimerization interface, which resembles that of related monooxygenases with a *para*-hydroxybenzoate hydroxylase (PHBH) fold (Figure 5b) (Montersino et al. 2013; Schreuder et al. 1988). Such mode of interaction seems advantageous for the binding of substrates and efficient catalysis, since in such a dimer the active sites point into the surrounding medium and provide space for the entering substrates. Especially, when reduced FAD is transferred from the reductase to the oxygenase component this might be beneficial to avoid auto-oxidation of FAD (Morrison et al. 2013). Interestingly, in the crystal structure of apo StyA from *Pseudomonas* sp. S12, the active sites of the dimer are located face to face (Figure 5a) (Ukaegbu et al. 2010). This orientation might not necessarily represent the active form since no data are available for the reduced protein. Thus, the structure of StyA1 with reduced FAD bound would be favorable to understand better the FAD transfer mechanism.

In StyA2B the StyB reductase is fused to the StyA2 oxygenase. Most StyB reductases occur as dimers (Gröning et al. 2014b; Morrison et al. 2013; Otto et al. 2004; van den Heuvel et al. 2004), and we also observe a dimeric nature for StyA2B and StyB from strain 1CP. From the apparent molecular mass determined by gel filtration (*M*_r_ = 111 ± 5 kDa; Figure 3; Table 3) we conclude that StyA2B has a globular fold. This strongly suggests that in one StyA2B subunit, two dimer interfaces (epoxidase and reductase domain) are present (Figure 5c, d) and that StyA2 forms a ‘double-back to back’ dimer.

**Table 3:** Hydrodynamic properties of StyA oxygenases from *R. opacus* 1CP as monitored by analytical gel filtration in the absence or presence of (reduced) FAD

StyA oxygenase	Elution volume (mL)^a^	Apparent molecular mass (kDa)^a^	Hydrodynamic state
StyA1	14.3	66	Monomer–Dimer
	14.0	75	Monomer–Dimer
	11.1	241	Oligomer
	8.2	≫	Polymer
StyA1 + FAD_ox_	14.3	66	Monomer–Dimer
	14.0	74	Monomer–Dimer
	10.8	273	Oligomer
	7.9	≫	Polymer
StyA1 + FAD_red_	13.8	81	Dimer
StyA2B	12.9	116	Dimer
	9.4	≫	Oligomer
	8.3	≫	Polymer
StyA2B + FAD_ox_	13.0	112	Dimer
	9.4	≫	Oligomer
	8.3	≫	Polymer
StyA2B + FAD_red_	13.1	107	Dimer
StyA	15.2	46	Monomer
	13.7	84	Dimer
	11.5	205	Tetramer
StyA + FAD_ox_	15.1	48	Monomer
	13.5	91	Dimer
StyA + FAD_red_	15.0	50	Monomer

^a^Superdex 200 column, bed volume = 22.0 mL. The mean of apparent molecular mass of repeated runs of protein samples is shown and the calculated standard deviation was between 5 and 10% in all cases.

The reason why the hydrodynamic state of investigated SMOs varies between the resting state and the active state is not simple to answer. Since we are dealing with isolated proteins, it could be that the heterologous produced StyA enzymes have a tendency to aggregate in the absence of their cofactor. On the other hand, the differences in oligomerization observed might point to an important regulatory mechanism, which still needs to be uncovered. It should be noted here that the possible interaction with the gel filtration material as well as the shape of the SMO proteins were not considered in this study. Additional studies from analytical ultracentrifugation or matrix-assisted light scattering might help to enlighten the aggregation behavior of the SMO proteins in further detail.

## Conclusion

In conclusion, the differences in hydrodynamic properties of StyA monooxygenases from strain 1CP depict additional evidence for a convergent evolution of these enzymes. Binding of (reduced) FAD to StyA enzymes inhibits their aggregation and results in either monomeric (StyA) or dimeric (StyA1, StyA2B) active forms. The present results suggest that incorporation of reduced FAD into StyA enzymes is attended with significant conformational rearrangements. Changes in protein–flavin interaction might also occur during catalysis, as previously observed for other monooxygenases with a PHBH fold (Huijbers et al. 2014; Montersino et al. 2011, 2013; Schreuder et al. 1988). More insight into the binding mode of reduced FAD and aromatic substrates is of utmost importance for understanding the catalytic potential and enantioselectivity of styrene monooxygenases.

## Additional files

**Additional file 1: In the Supplemental Material Section results from the protein purification and respective SDS-PAGE as well as the data from calibration runs for the analytical gel filtration are presented.**

## References

[CR1] Alonso S, Bartolomé-Martín D, del Álamo M, Díaz E, García JL, Perera J (2003). Genetic characterization of the styrene lower catabolic pathway of *Pseudomonas* sp. strain Y2. Gene.

[CR2] Anderson JL, Ding J, McCulla RD, Jenks WS, Armstrong DW (2002). Separation of racemic sulfoxides and sulfinate esters on four derivatized cyclodextrin chiral stationary phases using capillary gas chromatography. J Chromatogr A.

[CR3] Beltrametti F, Marconi AM, Bestetti G, Colombo C, Galli E, Ruzzi M (1997). Sequencing and functional analysis of styrene catabolism genes from *Pseudomonas fluorescens* ST. Appl Environ Microbiol.

[CR4] Gennaro PD, Colmegna A, Galli E, Sello G, Pelizzoni F, Bestetti G (1999). A new biocatalyst for production of optically pure aryl epoxides by styrene monooxygenase from *Pseudomonas fluorescens* ST. Appl Environ Microbiol.

[CR5] Gröning JAD, Eulberg D, Tischler D, Kaschabek SR, Schlömann M (2014). Gene redundancy of two-component (chloro)phenol hydroxylases in *Rhodococcus opacus* 1CP. FEMS Microbiol Lett.

[CR6] Gröning JAD, Kaschabek SR, Schlömann M, Tischler D (2014). A mechanistic study on SMOB-ADP1: an NADH:flavin oxidoreductase of the two-component styrene monooxygenase of *Acinetobacter baylyi* ADP1. Arch Microbiol.

[CR7] Hartmans S, van der Werf MJ, de Bont JAM (1990). Bacterial degradation of styrene involving a novel flavin adenine dinucleotide-dependent styrene monooxygenase. Appl Environ Microbiol.

[CR8] Hollmann F, Lin P-C, Witholt B, Schmid A (2003). Stereospecific biocatalytic epoxidation: the first example of direct regeneration of a FAD-dependent monooxygenase for catalysis. J Am Chem Soc.

[CR9] Huijbers MME, Montersino S, Westphal AH, Tischler D, van Berkel WJH (2014). Flavin dependent monooxygenases. Arch Biochem Biophys.

[CR10] Kantz A, Gassner GT (2011). Nature of the reaction intermediates in the flavin adenine dinucleotide-dependent epoxidation mechanism of styrene monooxygenase. Biochemistry.

[CR11] Kantz A, Chin F, Nallamothu N, Nguyen T, Gassner GT (2005). Mechanism of flavin transfer and oxygen activation by the two-component flavoenzyme styrene monooxygenase. Arch Biochem Biophys.

[CR12] Marconi AM, Beltrametti F, Bestetti G, Solinas F, Ruzzi M, Galli E (1996). Cloning and characterization of styrene catabolism genes from *Pseudomonas fluorescens* ST. Appl Environ Microbiol.

[CR13] Montersino S, Tischler D, Gassner GT, van Berkel WJH (2011). Catalytic and structural features of flavoprotein hydroxylases and epoxidases. Adv Synth Catal.

[CR14] Montersino S, Orru R, Barendregt A, Westphal AH, van Duijn E, Mattevi A (2013). Crystal structure of 3-hydroxybenzoate 6-hydroxylase uncovers lipid-assisted flavoprotein strategy for regioselective aromatic hydroxylation. J Biol Chem.

[CR15] Morrison E, Kantz A, Gassner GT, Sazinsky MH (2013). Structure and mechanism of styrene monooxygenase reductase: new insight into the FAD-transfer reaction. Biochemistry.

[CR16] Nikodinovic-Runic J, Coulombel L, Francuski D, Sharma ND, Boyd DR, Ferrall RMO (2013). The oxidation of alkylaryl sulfides and benzo[b]thiophenes by *Escherichia coli* cells expressing wild-type and engineered styrene monooxygenase from *Pseudomonas putida* CA-3. Appl Microbiol Biotechnol.

[CR17] O’Conner K, Buckley CM, Hartmans S, Dobson ADW (1995). Possible regulatory role for nonaromatic carbon sources in styrene degradation by *Pseudomonas putida* CA-3. Appl Environ Microbiol.

[CR18] Oelschlägel M, Zimmerling J, Schlӧmann M, Tischler D (2014). Styrene oxide isomerase of *Sphingopyxis* sp. Kp5.2. Microbiology (UK).

[CR19] Otto K, Hofstetter K, Röthlisberger M, Witholt B, Schmid A (2004). Biochemical characterization of StyAB from *Pseudomonas* sp. strain VLB120 as a two-component flavin-diffusible monooxygenase. J Bacteriol.

[CR20] Panke S, Witholt B, Schmid A, Wubbolts MG (1998). Towards a biocatalyst for (*S*)-styrene oxide production: characterization of the styrene degradation pathway of *Pseudomonas* sp. strain VLB120. Appl Environ Microbiol.

[CR21] Panke S, Wubbolts MG, Schmid A, Witholt B (2000). Production of enantiopure styrene oxide by recombinant *Escherichia coli* synthesizing a two-component styrene monooxygenase. Biotechnol Bioeng.

[CR22] Park MS, Bae JW, Han JH, Lee EY, Lee S-G, Park SH (2006). Characterization of styrene catabolic genes of *Pseudomonas putida* SN1 and construction of a recombinant *Escherichia coli* containing styrene monooxygenase gene for the production of (*S*)-styrene oxide. J Microbiol Biotechnol.

[CR23] Park J-B, Bühler B, Habicher T, Hauer B, Panke S, Witholt B (2006). The efficiency of recombinant *Escherichia coli* as biocatalyst for stereospecific epoxidation. Biotechnol Bioeng.

[CR24] Patrauchan MA, Florizone C, Dosanjh M, Mohn WW, Davies J, Eltis LD (2005). Catabolism of benzoate and phthalate in *Rhodococcus* sp. strain RHA1: redundancies and convergence. J Bacteriol.

[CR25] Schreuder HA, van der Laan JM, Hol WGJ, Drenth J (1988). Crystal structure of *p*-hydroxybenzoate hydroxylase complexed with its reaction product 3,4-dihydroxybenzoate. J Mol Biol.

[CR26] Thiel M, Kaschabek SR, Gröning JAD, Mau M, Schlömann M (2005). Two unusual chlorocatechol catabolic gene clusters in *Sphingomonas* sp. TFD44. Arch Microbiol.

[CR27] Tischler D, Eulberg D, Lakner S, Kaschabek SR, van Berkel WJH, Schlömann M (2009). Identification of a novel self-sufficient styrene monooxygenase from *Rhodococcus opacus* 1CP. J Bacteriol.

[CR28] Tischler D, Kermer R, Gröning JAD, Kaschabek SR, van Berkel WJH, Schlömann M (2010). StyA1 and StyA2B from *Rhodococcus opacus* 1CP: a multifunctional styrene monooxygenase system. J Bacteriol.

[CR29] Tischler D, Gröning JAD, Kaschabek SR, Schlömann M (2012). One-component styrene monooxygenases: an evolutionary view on a rare class of flavoproteins. Appl Biochem Biotechnol.

[CR30] Toda H, Itoh N (2012). Isolation and characterization of styrene metabolism genes from styrene-assimilating soil bacteria *Rhodococcus* sp. ST-5 and ST-10. J Biosci Bioeng.

[CR31] Toda H, Imae R, Komio T, Itoh N (2012). Expression and characterization of styrene monooxygenases of *Rhodococcus* sp. ST-5 and ST-10 for synthesizing enantiopure (*S*)-epoxides. Appl Microbiol Biotechnol.

[CR32] Ukaegbu UE, Kantz A, Beaton M, Gassner GT, Rosenzweig AC (2010). Structure and ligand binding properties of the epoxidase component of styrene monooxygenase. Biochemistry.

[CR33] van Berkel WJH, Kamerbeek NM, Fraaije MW (2006). Flavoprotein monooxygenases, a diverse class of oxidative biocatalysts. J Biotechnol.

[CR34] van den Heuvel RHH, Westphal AH, Heck AJR, Walsh MA, Rovida S, van Berkel WJH (2004). Structural studies on flavin reductase PheA2 reveal binding of NAD in an unusual folded conformation and support novel mechanism of action. J Biol Chem.

[CR35] van Hellemond EW, Janssen DB, Fraaije MW (2007). Discovery of a novel styrene monooxygenase originating from the metagenome. Appl Environ Microbiol.

[CR36] Velasco A, Alonso S, García JL, Perera J, Díaz E (1998). Genetic and functional analysis of the styrene catabolic cluster of *Pseudomonas* sp. strain Y2. J Bacteriol.

[CR37] Yeo Y-J, Shin S, Lee S-G, Park S, Jeong Y-J (2009). Production, purification, and characterization of soluble NADH-flavin oxidoreductase (StyB) from *Pseudomonas putida* SN1. J Microbiol Biotechnol.

