# Supplementary material for: Catalytic and hydrodynamic properties of styrene monooxygenases from Rhodococcus opacus 1CP are modulated by cofactor binding
Source: AMB Express. 2015 Jun 4;5:30. doi: 10.1186/s13568-015-0112-9 (PMC4460183; doi:10.1186/s13568-015-0112-9)
Supplement: Supplementary file 1 — Additional file 1: In the Supplemental Material Section results from the protein purification and respective SDS-PAGE as well as the data from calibration runs for the analytical gel filtration are presented. [file 13568_2015_112_MOESM1_ESM.pdf]

AMB Express

Catalytic and hydrodynamic properties of styrene monooxygenases from *Rhodococcus opacus* 1CP are modulated by cofactor binding

Anika Riedel,[a,b*] Thomas Heine,[a,b] Adrie H. Westphal,[b] Catleen Conrad,[a] Philipp Rathsack,[c] Willem J.H. van Berkel,[b] and Dirk Tischler [a,b*]

[a] Interdisciplinary Ecological Center, Freiberg Environmental Microbiology Group, TU Bergakademie Freiberg, Leipziger Str. 29, 09599 Freiberg, Germany

[b] Laboratory of Biochemistry, Wageningen University, Dreijenlaan 3, 6700ET Wageningen, The Netherlands

[c] Institute of Analytical Chemistry, TU Bergakademie Freiberg, Leipziger Str. 29, 09599 Freiberg, Germany

* Corresponding author. Mailing address: Environmental Microbiology, TU Bergakademie Freiberg, Leipziger Str. 29, 09599 Freiberg, Germany. Phone: 49-3731-394153. Fax: 49-3731-393012. E-mail: anika.riedel@ioez.tu-freiberg.de and dirk-tischler@email.de.

Supplemental Material Section

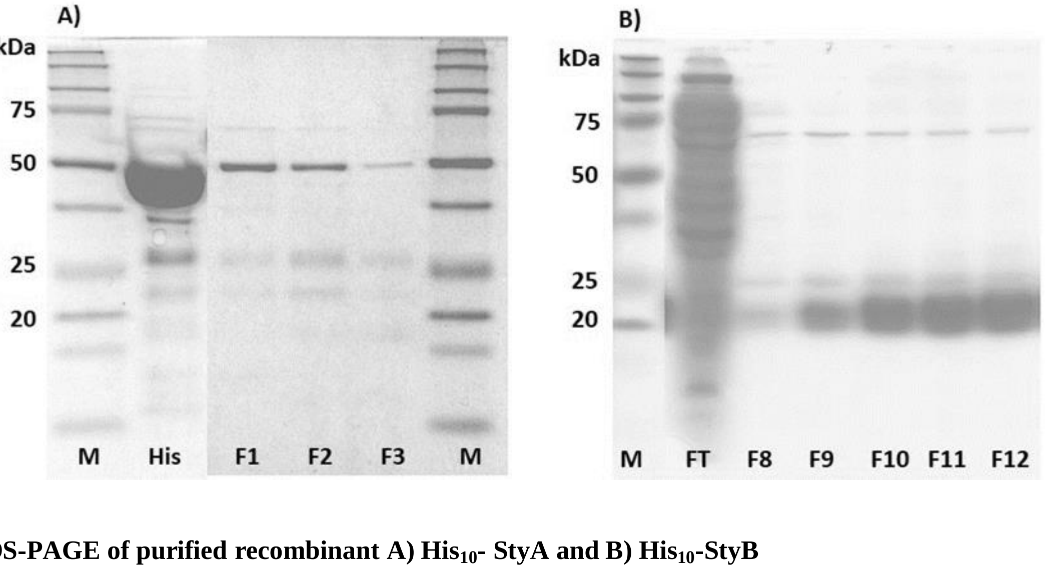

Fig. S1 SDS-PAGE of purified recombinant A) His_{10}- StyA and B) His_{10}-StyB

Proteins purified from crude cell extract via immobilized metal ion affinity chromatography (IMAC, **His**Trap FF column) and later applied to Superdex 200 column. **F1-F3:** Superdex fractions StyA at a size of 50 kDa. **F8-F12**: Superdex fractions StyB at a size of 21.5 kDa. **M** marker proteins: Precision Plus Protein Standard Dual Core (BioRad) 10-to 250-kDa. **FT** Flow-Through

Protein	MW [kDa]	Elution Volume [ml]	Kav
Cytochrome c	12.3	17.75	0.791
Myoglobin	17.8	17.33	0.757
alpha-Chymotrypsin	25.0	17.00	0.730
Ovalbumin	43.0	15.14	0.581
BSA-monomer	68.0	14.06	0.494
4-HO-benzoate-3-hydroxylase	90.0	14.14	0.501
Lipoamide dehydrogenase	102.0	13.47	0.447
BSA-dimer	136.0	12.58	0.376
Phenol-2-hydroxylase	152.0	12.14	0.340
Catalase	232.0	12.23	0.348
Ferritin	440.0	10.56	0.213
Vanillyl-alcohol oxidase	510.0	10.40	0.201

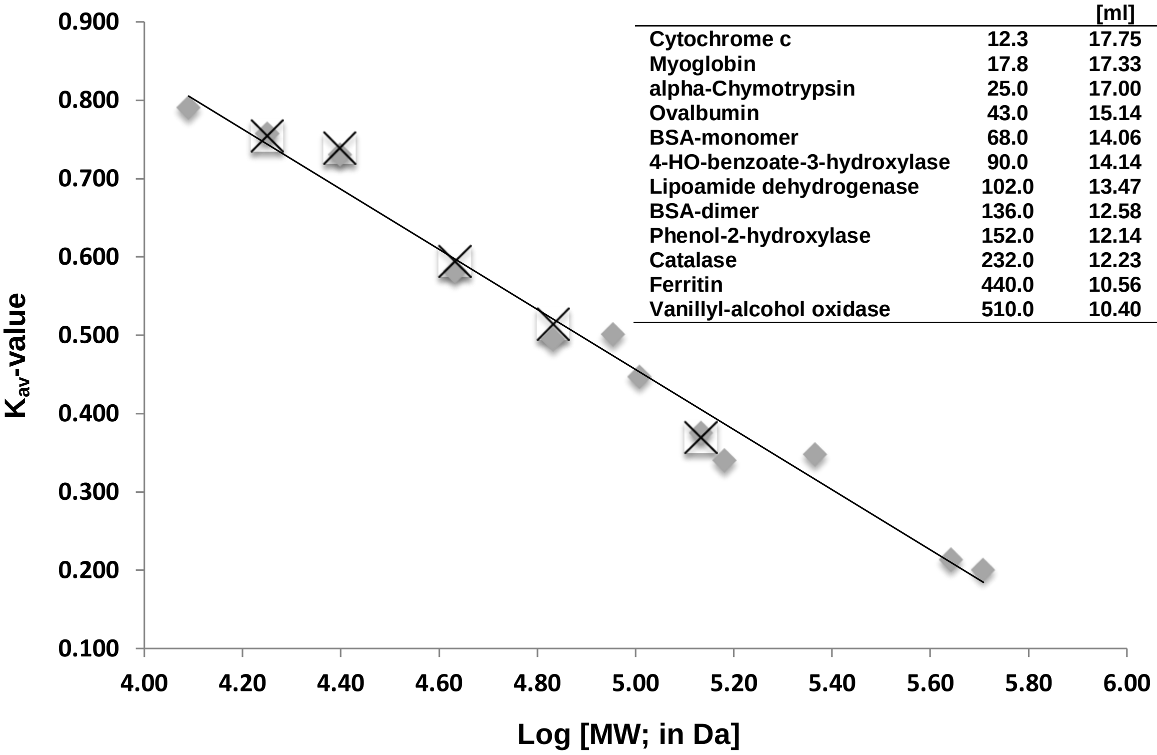

Fig. S2 Calibration of the applied Superdex 200 column for analytical gel filtration

Standards applied for calibration are given in the table. Standards were first analyzed prior the analytical runs with SMO samples (grey squares) and for some later again (black crosses). Calculations were performed as described in the manuscript (see equation 1)
